# A *Medicago truncatula* Metabolite Atlas Enables the Visualization of Differential Accumulation of Metabolites in Root Tissues

**DOI:** 10.3390/metabo11040238

**Published:** 2021-04-13

**Authors:** Clayton Kranawetter, Shuai Zeng, Trupti Joshi, Lloyd W. Sumner

**Affiliations:** 1 Department of Biochemistry, University of Missouri-Columbia, Columbia, MO 65211, USA; cdk374@mail.missouri.edu; 2 Metabolomics Center, University of Missouri-Columbia, Columbia, MO 65211, USA; 3 Christopher S. Bond Life Sciences Center, University of Missouri, Columbia, MO 65211, USA; zengs@mail.missouri.edu (S.Z.); joshitr@health.missouri.edu (T.J.); 4 Department of Electrical Engineering and Computer Science, University of Missouri, Columbia, MO 65211, USA; 5 MU Data Science and Informatics Institute, University of Missouri, Columbia, MO 65211, USA; 6 Department of Health Management and Informatics, School of Medicine, University of Missouri, Columbia, MO 65211, USA

**Keywords:** metabolomics, roots, rhizosphere, *Medicago truncatula*, border cells, Metabolite Atlas

## Abstract

Plant roots are composed of many differentiated tissue types, with each tissue exhibiting differential quantitative and qualitative accumulation of metabolites. The large-scale nontargeted metabolite profiles of these differentiated tissues are complex, which complicates the interpretation and development of hypotheses relative to the biological roles of differentially localized metabolites. Thus, we created a data visualization tool to aid in the visualization and understanding of differential metabolite accumulations in *Medicago truncatula* roots. This was achieved through the development of the *Medicago truncatula* Metabolite Atlas based upon an adaptation of the Arabidopsis Electronic Fluorescent Pictograph (eFP) Browser. *Medicago truncatula* roots were dissected into border cells, root cap, elongation zone, mature root, and root secretions. Each tissue was then analyzed by UHPLC-QTOF-MS and GC-Q-MS. Data were uploaded into a MySQL database and displayed in the *Medicago truncatula* Metabolite Atlas. The data revealed unique differential spatial localization of many metabolites, some of which are discussed here. Ultimately, the *Medicago truncatula* Metabolite Atlas compiles metabolite data into a singular, useful, and publicly available web-based tool that enables the visualization and understanding of differential metabolite accumulation and spatial localization.

## 1. Introduction

Multicellular eukaryotes have unique differentiated subcellular organelles, cells, and tissues that serve multiple biological and biochemical roles. These differentiated subcellular organelles, cells, and tissues further have unique physiology and metabolism. Thus, understanding the metabolic differences between unique cells and tissues provides biochemical insight into their unique function. We constructed a metabolite atlas to simplify the overall analysis of spatially differentiated metabolite accumulation. We tested the metabolite atlas by measuring and visualizing differential spatial accumulation of metabolites in roots of *Medicago truncatula*. The metabolite atlas enables the development of hypotheses related to the biological importance of metabolite spatial accumulation. Of particular interest to our laboratory is understanding the metabolites involved in mediating plant–microbe interactions. The dynamics of these interactions are poorly understood and in need of further research.

Broadly speaking, plant roots are composed of mature tissue, the elongation zone, and the root cap. The root cap consists of several other cell types including stem cells, columella cells, lateral root cap cells, and border cells. Biotic or abiotic damage to the root cap can be disastrous for the plant as it can result in termination of root growth and/or ultimately death of the plant [1,2].

Many plants defend the root cap through secretions and special cells termed border cells [3]. Border cells are an often-overlooked part of root studies and their considerable contributions to rhizosphere dynamics are, as such, not considered or properly attributed [4,5]. Furthermore, these contributions cannot be properly assessed through studies utilizing the popular model system *Arabidopsis thaliana* as this plant species, along with other plant species from the *Brassicaceae* family, do not possess border cells but instead possess border-like cells [6]. Border-like cells are morphologically distinct and, thus far, have demonstrated only a few shared functions with border cells [7,8]. However, the focus of this report will be on traditional border cells that are found on most other plant species.

Once cells reach the outermost part of the root epidermal layer, pectin methylesterases are activated, resulting in a breakdown of the cell walls connecting border cells to the root tip [9,10,11]. This results in the release of border cells as single cells or small clumps of cells. After their release, border cells remain tightly appressed to the root tip through a self-secreted mucilage [12,13,14,15]. This secreted mucilage can support microbial growth, potentially serving as a chemoattractant, and plays a role in trapping of toxic metals such as lead and aluminum [13,14,16]. As border cells are contained within a water-soluble matrix, they disperse easily in water. After removal, border cells are rapidly replaced within 24 h, with the total number of cells being species specific [9,12].

Border cells are major secretory cells, with some of their secreted components directly mediating host–microbe interactions [17,18]. A major secreted component is DNA that tangles together to form a physical “net”-like structure that entangles pathogenic microbes [19,20]. Removal of this DNA abolishes host defense against invading fungal pathogens [17]. Furthermore, it has been shown that various microbes possess extracellular DNase activity, allowing them to escape from extracellular DNA structures [18,21]. This mechanism is similar to that of mammalian, DNA-based neutrophil extracellular traps (NETs) [22].

Border cells also secrete a multitude of proteins and metabolites [4,7]. Upon their release from the root tip, there is large-scale genetic reprogramming of border cells [4,23]. Border cells contain starch reserves which serve as an energy source, which can be diverted from primary metabolism and growth to the biosynthesis of specialized (also known as secondary) metabolites during biotic and abiotic stress [4]. Furthermore, reprogramming also results in newly produced proteins, many of which can be found to be rapidly secreted to the extracellular environment [23].

While border cells have been shown to possess and secrete a multitude of metabolites, the biological context of these metabolites is poorly understood. This lack of understanding is further complicated by both the challenge of confident metabolite identification and the lack of sufficient spatial resolution to assign border cell specific metabolites. This makes it difficult to understand the roles of secreted plant metabolites. Current metabolite identifications at the existing resolution hint at potentially powerful plant-rhizosphere interactions [4]. A caveat with this existing data is the lack of separation of border cells from the secreted component. As secreted metabolites are those that would influence rhizosphere chemistry, it is better to separate and characterize these metabolites independently. This ultimately provides the necessary spatial resolution to develop better hypotheses related to metabolites localized in the secreted component.

Large-scale nontargeted metabolomics experiments, such as the one in this current study, often contain many compounds, not all of which are currently identified. The sheer scope and complexity of this data would greatly benefit from more effective means of data visualization and interpretation. To achieve this benefit, a Metabolite Atlas for *Medicago truncatula* roots was developed using the Arabidopsis Electronic Florescent Pictograph (eFP) browser software [24]. Formation of the *Medicago truncatula* Metabolite Atlas was achieved utilizing the Arabidopsis eFP browser background calculation infrastructure paired with a redeveloped web interface and tissue imagery representative of *M. truncatula*. The eFP Browser software was originally developed to visualize heat maps of gene expression across multiple tissue types in *Arabidopsis thaliana*. A particularly attractive aspect of this tool is its use of tissue representative pictures to display tissue-specific gene expression data. Data displayed in such a format is more easily visualized and improves clarity by using tissue representative images (e.g., using a leaf to show leaf-related data). This overall structure can also be utilized for displaying metabolite spatial localization by tissue type. It makes metabolomics data more easily interpretable while retaining data specificity.

In this study, we discuss the customization and modifications of the Arabidopsis eFP Browser to display metabolite spatial localization in our newly formed *Medicago truncatula* Metabolite Atlas. In addition to, this, we used the *Medicago truncatula* Metabolite atlas to compare metabolite spatial localization within border cells, the root cap, the elongation zone, mature tissues, and extracellular secretions from roots of *Medicago truncatula*.

## 2. Results

### 2.1. Data Generation and Compilation in the M. truncatula Metabolite Atlas

**Figure 1:**
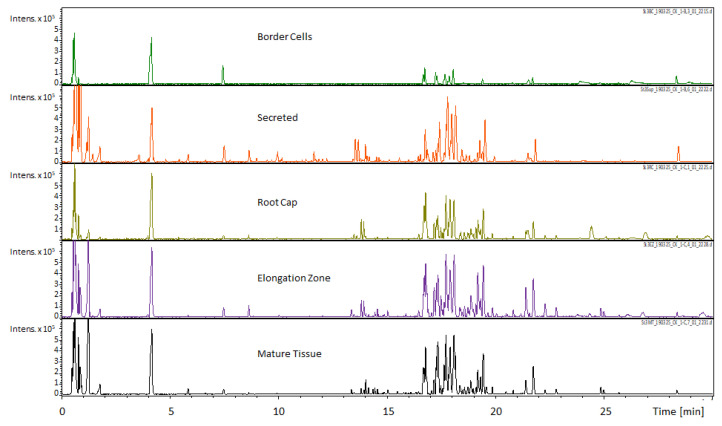
Comparison of UHPLC-MS chromatograms obtained for different *M. truncatula* root tissues (scale ×10^5^). The Umbelliferone internal standard can be seen immediately after the 4 min time point. Observational comparison of the five sampled regions revealed differential accumulation of metabolites with the most readily apparent differences in the 0 to 2 min and 16 to 19 min time span. These qualitative data indicate differential accumulation of metabolites that can potentially be linked to specialized functions.

*Medicago truncatula* roots were dissected into border cells, the root cap, the elongation zone, mature tissue, and secretions. For this paper, the term secretions are defined as those extracellular compounds that are outside of the root/border cells and would typically be released into the rhizosphere. Dissected tissues were extracted and analyzed by UHPLC–QTOF–MS and GC–Q-MS. These data were compiled and loaded into a MySQL database table that was utilized in formation of the *Medicago truncatula* Metabolite Atlas. Visual inspection of resultant metabolite profiles showed key differences between individual root components (Figure 1). The border cell component was notably lacking in metabolite diversity and overall intensity. Conversely, visual inspection indicated that the other analyzed components exhibited a larger metabolite diversity and intensity compared to border cells. Results from both GC-MS and UHPLC–MS analyses of root regions reveal lower metabolite intensity and diversity in border cells compared to all other fractions. Thus, our UHPLC–MS and GC-MS data showed that border cells possess fewer internal metabolites compared to the other components analyzed.

**Figure 2:**
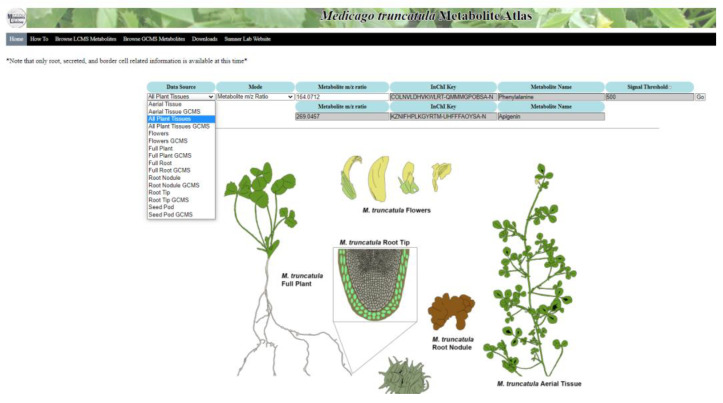
Home page of the *Medicago truncatula* Metabolite Atlas. This atlas provides multiple plant tissues for visualization of metabolite localization. Ultimately, this metabolite atlas is a useful tool showing metabolite localization in a way that can be easily conveyed to users.

A customized installation of the Arabidopsis Electronic Fluorescent Pictograph (eFP) Browser was used for the creation of the *Medicago truncatula* Metabolite Atlas (Figure 2). The background calculation infrastructure was modified to incorporate different nomenclatures and data types. All other background infrastructure was kept the same as the original in the Arabidopsis eFP browser. The visible webpage interface was also extensively redeveloped, with all *Arabidopsis* related imagery removed and replaced with *M. truncatula* relevant imagery. The root tip specific image showing border cells was modified with permission from an original illustration to show the secreted metabolite component [25]. Other images were added to show all tissues of *M. truncatula* (which we termed all plant tissues) and included a complete individual *M. truncatula* plant, aerial tissue, flowers, root nodules, root tip, full root tissues, and a *M. truncatula* seed pod. The data source selection menu features a drop-down list of all tissue types that are contained within the atlas (Figure 2). Currently, only root-related data are available, but others can/will be added in the future. The data sources were divided such that UHPLC–MS data and GC-MS data remain separate.

The homepage image was color coded to illustrate the different tissue regions. The chosen colors for the starting image have a specific hex-code, provided to the background programming, that serves as a reference for the program. These reference hex-codes are selected and then changed to reflect relative metabolite intensity upon entering a search query. This results in the same heat map format utilized in the Arabidopsis eFP browser, but reflects relative metabolite intensity rather than gene expression. The same intensity scaling was utilized from the Arabidopsis eFP browser, with lighter shades (yellow) indicating lower intensity and darker shades (red) indicating higher intensity. Shades of grey indicate the absence of a compound. Mass-to-charge ratio or International Chemistry Identifier (InChI) Key searches are available and result in a database check, attainment of the query intensity level, and charting of the intensity level based on the color schematic. A chart showing this color schematic and the associated relative intensity numbers is displayed along with the heat map image.

The MySQL database information currently includes metabolite m/z ratios, names, formulas, InChI keys, and intensity levels for both UHPLC-MS and GC-MS data. Known compounds were assigned a formal InChI key, while unknown compounds were assigned a working code based upon proposed standardized nomenclature guidelines [26]. Both the formal InChI Key and working code can be utilized to search for a given compound. The pages listing mass features contained within the atlas are designed such that clicking on a m/z ratio will automatically search for the chosen m/z ratio using the All Plant Tissues page.

### 2.2. UHPLC-MS Analyses of Root Sections Show Differential Metabolite Accumulation

**Figure 3:**
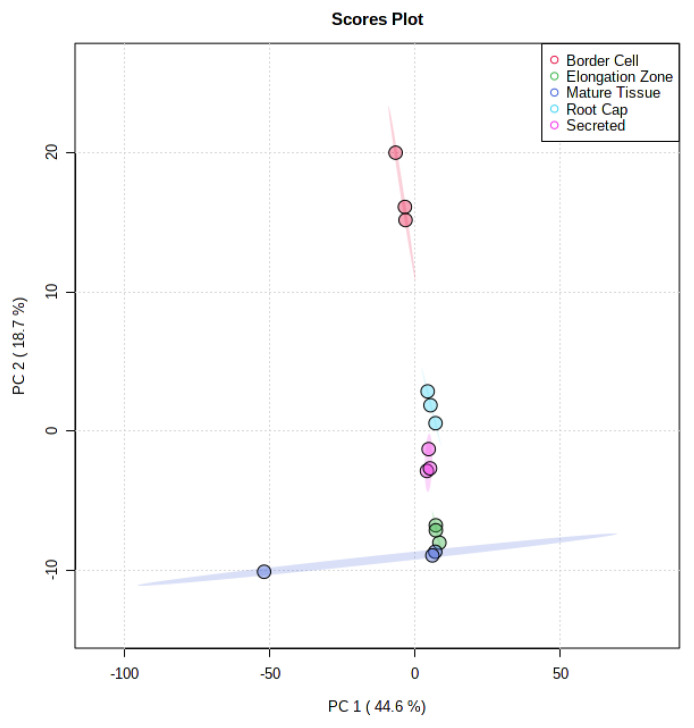
Principle Component Analysis of UHPLC-MS Analyzed Fractions. Comparison of all five fractions indicates a 44.6% variance in the first principle component and 18.7% variance in the second component. The spatial separation of fractions indicates differential localization of metabolites.

Differential accumulation of metabolites between spatially separated components were observed utilizing UHPLC-QTOF-MS. A total of 511 mass features were detected, with 246 of these features possessing available MS/MS data. Of these, 42 compounds were identified, with 14 MS1, 17 MS2, and 11 MS3 level identifications according to established metabolomics reporting standards [27]. The remaining features were not identified and assigned MS4 level status according to established metabolomics reporting standards [27]. Comparison of metabolite profiles using Principle Component Analysis (PCA) revealed substantial separations between each tissue (Figure 3). Principle component one (PC1) accounted for 44.6% of differences seen between fractions, while principle component two accounted for 18.7% of differences seen between fractions. There appeared to be some overlap between these tissues, which is to be expected given the possibility of metabolites being present within more than one tissue.

**Figure 4:**
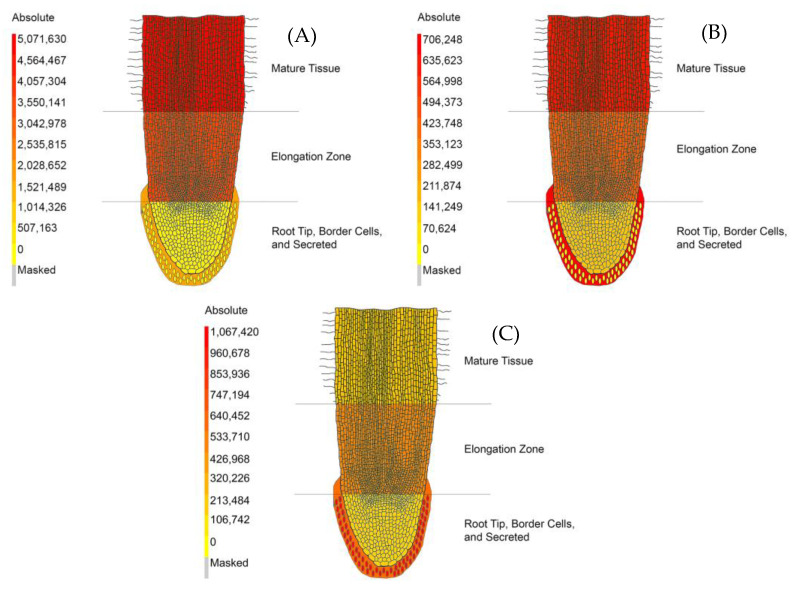
The *Medicago truncatula* Metabolite Atlas Spatial Localization of UHPLC–MS Analyzed Metabolites. (**A**) Relative spatial localization of phenylalanine. (**B**) Relative spatial localization of tryptophan. (**C**) Relative spatial localization of 7,4′-dihydroxyflavone. These data indicate variances in spatial localization of certain metabolites while showing them in a format that can be widely and easily understood.

There were several notable metabolites that displayed differential localization. For example, the amino acid phenylalanine was found to vary in intensity across individual root tissues. The mature tissue and elongation zone fractions had the highest relative levels of phenylalanine and have visually comparable intensities (Figure 4A). Further comparison calculations by region showed a 1.34-fold increase in phenylalanine in mature tissue (average 5,071,633 counts with 6% RSD) compared to the elongation zone (average 3,777,227 counts with 6% RSD) (Supplementary Materials Table S1). The secreted fraction showed a relative intensity of 0.37-fold (average 1,856,525 with 16% RSD) that of the mature tissue and 0.49 of the elongation zone fractions. The root cap (average 385,426 counts with 15% RSD) and border cells (41,030 with 26% RSD) possessed a comparatively smaller amount of phenylalanine, with border cells possessing the lowest relative amount. An interesting observation was the somewhat higher level of phenylalanine in the secreted fraction, with a 45.25-fold increase in the secreted component compared to border cells (Supplementary Materials Table S1).

Another amino acid that was found to vary across fractions was tryptophan (Figure 4B). The intensity of tryptophan in border cells (average 13,015 counts with 25% RSD) was found to be effectively absent compared to that of the secreted component (average 706,248 counts with 19% RSD) and the root cap (average 174,104 counts with 11% RSD) (Supplementary Materials Table S1). The secreted component possessed the highest intensity of tryptophan with a 4.06-fold increase compared to the root cap, a 1.53-fold increase compared to the elongation zone (average 462,221 counts with 14% RSD), and a 1.10-fold increase compared to mature tissue (average 640,619 counts with 12% RSD) (Supplementary Materials Table S1).

The intensity of 7,4′-dihydroxyflavone (DHF) indicated spatial localization between the five regions analyzed in our experiments (Figure 4C). DHF was seen at its highest intensity in border cells (average 1,067,422 counts with 11% RSD) with a 1.72-fold increase compared to the secreted component (average 618,915 counts with 32% RSD), which had the second highest intensity of DHF (Supplementary Materials Table S1). This compound was at a lower intensity in the root cap (average 205,029 counts with 6% RSD), the elongation zone (average 521,559 counts with 51% RSD), and mature tissue (average 260,451 with 19% RSD). DHF levels in border cells had a 5.21-fold increase compared to the root cap, a 2.05-fold increase compared to the elongation zone, and a 4.10-fold increase compared to mature tissue (Supplementary Materials Table S1). The metabolites mentioned here illustrate the functionality of the metabolite atlas. Other metabolites and differential metabolite accumulation can be further visualized using the *Medicago truncatula* metabolite atlas tool. A full list of metabolomics standard initiative (MSI) compliant identified and unidentified compounds has also been compiled for in depth analysis (Supplementary Materials Table S1) [27].

### 2.3. GC-MS Analyses of Root Sections Show Differential Accumulation of Metabolites

**Figure 5:**
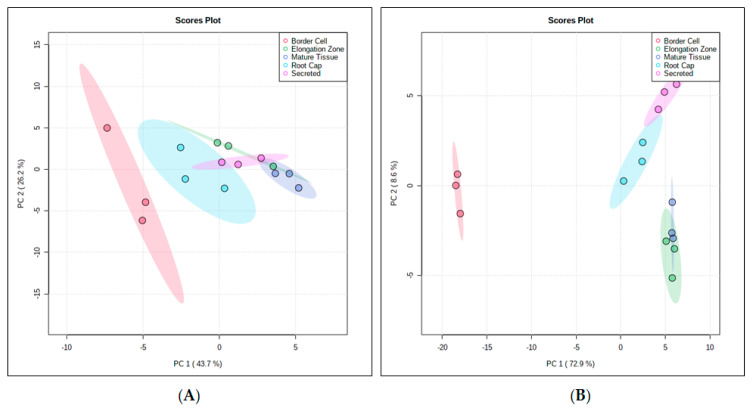
PCA Analyses of GC-MS polar (**A**) and GC-MS nonpolar (**B**) *Medicago truncatula* Root Fractions. PCA analysis of the GC-MS polar data shows a separation between fractions, with border cells separating away from other fractions. Nonpolar GC-MS analysis also showed separation between fractions, with border cells again separating away from other fractions. For both analyses, root tissue fractions showed less separation. These data illustrate the differential accumulation or spatial localization of metabolites within unique root tissues.

Nontargeted GC-MS metabolomics data were collected for the various differentiated root tissues. GC-MS polar analysis resulted in 377 mass features with 103 identified metabolites. Of these identified metabolites, there were 63 MS1, 12 MS2, and 28 MS3 assigned identifications according to established metabolites reporting guidelines [27]. All remaining mass features were unknown and assigned MS4 status [27]. GC-MS nonpolar experiments resulted in 127 mass features with 24 identified metabolites. Of the identified metabolites, 21 were assigned MS1 status, with no MS2 level identifications and three MS3 according to established metabolomics reporting guidelines [27]. All remaining mass features were unknown and assigned MS4 status [27]. PCA of the GC-MS polar extract data indicated that principle component one accounted for 43.7% of differences seen between fractions, while principle component two accounted for 26.2% of differences seen between fractions (Figure 5A). For GC-MS nonpolar extract data, principle component one accounted for 72.9% of differences seen between fractions, while principle component two accounted for 8.6% of the difference seen between fractions (Figure 5B).

Multiple other metabolites were observed to vary greatly between components. These metabolites were chosen to illustrate functionality of the metabolite atlas, but are not indicative of global metabolite changes. Specific data values, metadata, and the full list of MSI compliant identified and unidentified compounds have been compiled for further analysis (Supplementary Materials Table S2) [27].

Malic acid was observed at a higher intensity in the border cells (15,961 counts with 118% RSD) but indicated a high degree of variability with wide RSD values (Supplementary Materials Figure S1A and Table S2). The secreted fraction possessed the next highest intensity (average 5607 with 32% RSD) (Supplementary Materials Figure S1A and Table S2). The next highest appears in the elongation zone (average 3790 counts with 29% RSD, respectively) and root cap fraction (average 2861 counts with 63% RSD, respectively (Supplementary Materials Figure S1A and Table S2). The lowest intensity of malic acid was seen in the mature tissue fraction (average 755 counts with 29% RSD, respectively) (Supplementary Materials Figure S1A and Table S2).

Citric acid was seen at higher levels in the border cell fraction (average 1438 counts with 106% RSD) relative to other fractions, but possessed a very high variance (Supplementary Materials Figure S1B and Table S2). The secreted fraction possessed the next highest intensity (average 516 counts with 37% RSD) followed by the root cap (average 318 counts with 50% RSD), the elongation zone (average 242 counts with 7% RSD), and mature tissue (average 88 counts with 41% RSD) (Supplementary Materials Figure S1B and Table S2).

Succinic acid was also observed at its highest level in the border cell region (average 4726 counts with 99% RSD), but again, possessed a high RSD (Supplementary Materials Figure S1C and Table S2). The border cell fraction was followed by the secreted fraction (average 1518 counts with 72% RSD) with border cells possessing a 3.11-fold increase compared to the secreted component (Supplementary Materials Figure S1C and Table S2). The root cap (average 505 counts with 56% RSD), the elongation zone (average 430 counts with 6% RSD), and mature tissue (average 132 counts with 52% RSD) fractions had decreasing levels of succinic acid, respectively (Supplementary Figure S1C and Table S2). The root cap possessed 1.18-fold more succinic acid than the elongation zone, which possesses 3.26-fold more succinic acid than the mature tissue region (Supplementary Materials Figure S1C and Table S2).

## 3. Discussion

### 3.1. Visualization of Metabolite Spatial Localization Is Improved Using the Medicago truncatula Metabolite Atlas

The compilation of data into a metabolite atlas provides a repository for easy public access to data, enhances data interpretation, and ultimately facilitates future hypothesis development. Using a heatmap associated with a tissue image display simplifies the process of exhibiting spatial localization data. Provision of such data in numerical formats and application of statistical methods are often valuable and necessary, but can be cumbersome when trying to grasp widespread changes. A metabolite atlas and heat map system help enhance understanding of how metabolite intensities are changing between tissues before focusing on specific numerical values and statistical methods.

With the ever-increasing presence of big data studies, data display and visualization tools become a necessity rather than a luxury. Here, we introduce the *Medicago truncatula* Metabolite Atlas for visualization of metabolomics data. The *Medicago truncatula* Metabolite Atlas was initially populated with root specific metabolomics data. The *Medicago truncatula* Metabolite Atlas can be expanded in the future to include spatial localization on a broader range of tissue types (such as aerial tissues, flowers, and root nodules), expansion of search refinement capabilities, and incorporation of tools for in-depth exploration of metabolite profiles if sufficient public interest and financial support can be obtained.

### 3.2. UHPLC-MS and GC-MS Analyses Reveal Spatial Localization of Potentially Significant Metabolites

Previously, we compared the transcriptome analyses of border cells to the root tip and increases in secondary metabolism transcripts associated with phenylpropanoids and phenolic biosynthesis were observed [4]. Data published by Watson et al. collected the secreted component and border cells together as one fraction [4]. In this report, border cells and the secreted components were separated and revealed that phenylalanine is more abundant in the secreted component rather than intracellular levels in border cells. It is also possible that phenylalanine is synthesized in border cells and secreted, either actively or passively, into the rhizosphere environment. This is supported by previous transcriptomics data that indicated increases in border cell intracellular enzymes associated with phenylalanine metabolism [4]. Chorismate is a precursor in phenylalanine biosynthesis and is converted to prephenate by chorismate mutase (Supplementary Materials Figure S2). Transcripts associated with chorismate mutase were seen to be expressed at a level 4.1-fold higher in border cells than that of root tips [4]. Prephenate can then be converted to l-arogenate, 4-hydroxy-phenylpyruvate, or phenylpyruvate by prephenate aminotransferase, prephenate dehydrogenase, or prephenate dehydratase, respectively [28]. Transcripts associated with prephenate dehydratase were seen to be 2.2-fold higher in border cells compared to the root tip [4]. The result of this would be an increase in phenylpyruvate which is converted into phenylalanine through the action of an aminotransferase. Previously, a phenylpyruvate aminotransferase from a class of tyrosine aminotransferases was identified which relied on tyrosine and phenylpyruvate for transamination and ultimately formation of phenylalanine [28]. A partially matched (88%) transcript associated with tyrosine aminotransferases was 4.1-fold higher in border cells relative to root tips, potentially representing this phenylpyruvate aminotransferase [4]. The pathway utilizing phenylpyruvate to produce phenylalanine has been shown to be a viable alternative to the traditional pathway using arogenate [28]. One major difference in these two pathways for phenylalanine biosynthesis is the subcellular localization. Typical phenylalanine biosynthesis occurs within plastids but production of phenylalanine through phenylpyruvate occurs in the cytosol [28]. Cytosolic biosynthesis of phenylalanine, much like the plastid bound pathway, is linked to production of phenylalanine derived volatile compounds [29]. This function matches with the previously established role of border cells wherein volatile compounds would be emitted in a defense-related capacity, adding to the plethora of other defense-related properties attributable to border cells [4]. This seems likely considering emission of hexenal, a volatile compound possessing defense-related properties, is increased in border cells relative to other root tissues [4]. With soil volatile compounds known to play a role in plant-plant and plant–microbe signaling, it is not a stretch to include border cells as a possible source of volatile signaling-related compounds [30,31].

Our combined data lead us to hypothesize that phenylalanine is synthesized in border cells and secreted into the extracellular environment. Phenylalanine is utilized in biosynthesis of a multitude of cellular components and compounds such as proteins, lignin, phenylpropanoids, flavonoids, anthocyanins, and hormones [29]. The purpose of secreted phenylalanine is not clear beyond serving as a potential chemoattractant and possibly supporting microbial growth.

The variation in tryptophan levels amongst the root tissues supported the hypothesis that border cells secrete metabolites that serve as chemoattractants. The pathway to tryptophan biosynthesis begins, like phenylalanine, with chorismate (Supplementary Materials Figure S2). In tryptophan biosynthesis, chorismate is converted to anthranilate in a committed step by anthranilate synthase (Supplementary Materials Figure S2). Previous transcriptomics data showed anthranilate synthase transcripts to be 2.6-fold higher in border cells compared to the root tip [4]. Other transcripts associated with the tryptophan biosynthetic pathway were noticeably absent. The transcript associated with tryptophan synthase, the last enzyme in the tryptophan biosynthetic pathway, was observed at higher levels in the root [4]. It is important to note that the root tip region data in the Watson et al. article included the root tip as the terminal 2–4 mm of the root [4]. This would include both the apical meristematic region and part of the elongation zone. With the border cells and terminal 1–2 mm of the root containing inconclusive transcriptomics data on tryptophan biosynthetic localization, multiple possibilities arise. With border cell tryptophan associated transcripts showing some level of increased expression, it seems that at least some of the tryptophan in the secreted fraction could be arising from the border cells. As noted previously, border cells have been shown to be major secretory cells, this is a plausible hypothesis that is worthy of further testing by specifically targeting transcripts and enzymes associated with tryptophan synthesis and transport. Some of the secreted tryptophan may also be arising from the root tip/elongation zone considering the increased expression of tryptophan biosynthesis associated transcripts in the root.

We propose a mechanism involving multiple sources of secreted tryptophan while maintaining a localization in specific root tissues. Secreted tryptophan has been shown to serve as a chemoattractant for nodulating bacterium *Rhizobium melliloti* [32]. This bacterium detects soil tryptophan and converts it to indole acetic acid (IAA), which is a plant hormone that plays a role in root growth and nodulation [33]. The elongation zone possessing a higher intensity of tryptophan would likely attract soil nodulating microbes to this region over regions possessing lower levels of tryptophan. We hypothesize this would place nodulating microbes in a prime location for initiation of nodulation as the root continues to grow and root hairs form. Nodulation begins at the site of a root hair in the mature tissue region [34]. Attraction of nodulating microbes to the elongation zone would place them in the proper location for initiation of nodulation upon future root hair development.

The role of 7,4′-dihyroxyflavone (DHF) is highly characteristic of border cell functionality as it serves in both defense and commensal roles. Previous characterization provided a large-scale view that the secreted environment and border cells combined had more DHF [4]. The increased spatial resolution in our experiments show a more complete story wherein DHF is localized not just in border cells but also in the secreted component (Figure 4C). It is likely that DHF is arising mostly from border cells. In our discussion on phenylalanine above, we showed that there was an increase in phenylalanine synthesis in border cells. This phenylalanine can be shuttled into phenylpropanoid biosynthesis where phenylalanine is converted to cinnamic acid by phenylalanine ammonia lyase (PAL) (Supplementaty Materials Figure S3). Previous data showed transcripts associated with PAL were increased 16.2-fold in border cells relative to the root tip [4]. Cinnamic acid is then converted into cinnamoyl-CoA by 4-coumarate-CoA ligase (Supplementary Materials Figure S3). Trans-cinnamate 4-monooxygenase then converts cinnamoyl-CoA into p-coumaroyl-CoA (Supplementary Materials Figure S3). P-coumaroyl-CoA is then converted into isoliquiritigenin by chalcone synthase (Supplementary Materials Figure S3). Border cells were shown to have a 50.3-fold increase in chalcone synthase relative to the root tip [4]. Isoliquiritigenin is converted into liquiritigenin by chalcone isomerase, which shows a 2.0-fold increase in border cell transcripts relative to the root tip (Supplementary Materials Figure S3) [4]. Finally, liquiritigenin is converted into DHF by flavone synthase I/II (Supplementary Materials Figure S3). Flavone synthase was seen to have an 8.1-fold increase in transcripts relative to the root tip and 18.1-fold relative to the whole root [4]. These transcriptomics data combined with our spatial localization data reveal border cells as heavy producers and secretion machines of DHF.

DHF has been shown to serve a multitude of functions that provide insight into the utility of its spatial localization in border cells and the secreted component. DHF has been shown to serve as a moderate inducer of *nod* genes in *Rhizobium meliloti*, potentially helping to facilitate initiation of nodulation as a secreted metabolite [35,36]. Conversely, DHF is secreted in response to elicitation by extracts from the fungal pathogen cotton root rot (*Phymatotrichopsis omnivora*) [4]. This indicates a duality in the possible function of DHF as it could play a role, as a secreted metabolite, in attraction of nodulating microbes. However, the relatively high spatial localization and previous data indicating DHF secretion as a response to fungal pathogen elicitation hints at this compound being held in border cells to be used as an antimicrobial defense mechanism.

Malic acid was observed at a higher relative abundance in border cells and the secreted fraction. Previous transcriptomics data showed a 46.4-fold increase in malate synthase compared to the root tip, which interconverts glyoxylate and malate [4]. The split between border cells and the secreted fraction, along with the increase in a transcript associated with malic acid synthesis, indicates this metabolite is secreted by border cells. Malic acid plays a role in a multitude of cellular processes, including the glyoxylate and citric acid cycles. The previously shown decrease in border cell fumarate hydratase transcripts, which converts fumarate to malate in the citric acid cycle, indicates malic acid as being mostly glyoxylate cycle produced in the case of border cells [4]. As a secreted compound malic acid has been shown in *Arabidopsis thaliana* to be involved in recruitment of beneficial microbes, potentially serving as a chemoattractant metabolite [37]. Malic acid is also associated with aluminum tolerance and serves as a dicarboxylic acid nutritional source for bacteria [38,39].

Citric acid was also observed to be primarily localized in border cells and the secreted component. Previous data indicated that it was border cells that have increased citrate, but our data indicates that citric acid was not just localized in border cells but was also high in the secreted fraction [4]. Border cells showed the highest level of citric acid of all regions and this, combined with previously published data, implies that border cells are the primary source of synthesis and secretion of citric acid [4]. This has important implications in involvement of border cells in rhizosphere deposition of organic acids. As previously mentioned, malic acid seems to be present at a higher intensity in the secreted fraction than in other fractions. Secretion of organic acids, such as malic and citric acid, is associated with mobilization of insoluble, soil-bound elemental nutrients for root uptake [40].

Similarly, succinic acid was observed primarily in the border cell and secreted fractions. As an intermediate in the citric acid cycle, it represents a possible mobilization of energy stores within border cells. It participates in a multitude of pathways, making it a rather diverse compound. As a secreted organic acid, it has the potential to play a role in attraction of commensal microbes and mobilization of immobile nutrients for plant root uptake [39,40,41].

## 4. Materials and Methods

### 4.1. Tissue Collection

*Medicago truncatula* seeds were harvested and chemically scarified according to an established protocol [42]. Scarified seeds were germinated in Petri dishes on sterile filter paper overlaying 1% w/v water agar (PhytoTech Labs, Lenexa, KS, USA) at 23 °C in the dark for three days (72 h). Sample sets were prepared in triplicate with each set containing a total of 45 seedlings. Seedlings were maintained in the dark during tissue collection by wrapping Petri dishes in aluminum foil. Border cell and secreted metabolite samples were collected by dipping root tips into sterile water and lightly vortexing for 1 min. These samples were maintained in water on ice during collection. Border cells were separated from the secreted component by centrifugation at 500× *g* for 10 min and removal of supernatant to a clean tube. After removal of border cells and secreted metabolites, root sections were defined by observation under a Leica-M205-Stereomicroscope (Leica Microsystems, Wetzlar, Germany). The root cap sample was defined as the region containing the meristematic zone and the root tip, the elongation zone as the area behind the meristematic zone prior to root hair development, and mature tissue as the area containing root hairs. Each zone was harvested through manual excision using a #11 scalpel. Excised tissues were immediately placed in 40% methanol: 60% sterile Milli-Q water and maintained on ice to prevent further metabolic activity. All samples were flash frozen in liquid nitrogen after tissue collection was completed. All samples were lyophilized for 48 h to remove residual water and methanol.

### 4.2. UHPLC-ESI-QTOF-MS

Lyophilized samples were subjected to metabolite extraction in 80% methanol: 20% LC–MS Grade H_2_O, maintaining a 6 mg of dry tissue per 1 mL concentration. Extraction was performed for 2 h at 4 °C using vigorous agitation on an orbital shaker. Sample extracts were centrifuged at 15,000× *g* for 10 min to pellet debris and supernatant was removed to a clean tube. Sample extracts were dried under nitrogen gas flow at 30 °C and resuspended in 80% methanol: 20% LC-MS-grade H_2_O containing 6 µg/mL umbelliferone internal standard to obtain a final concentration of 50 µL/mg of starting dry tissue. All samples were centrifuged a final time at 15,000× *g* for 10 min to pellet debris before removing the supernatant to autosampler vials.

Sample analyses were performed using a Bruker Impact II quadrupole-time-of-flight mass spectrometer coupled to a Waters ACQUITY UHPLC system. Chromatographic separations were performed using a Waters C18 column (2.1 × 150 mm, BEH C18 with 1.7-µm particles), a linear gradient composed of mobile phase A (0.1% formic acid) and B (Acetonitrile). Gradient conditions: B increased from 5% to 70% over 30 min, then to 95% over 3 min, held at 95% for 3 min, then returned to 5% for equilibrium. The flow rate was maintained at 0.56 mL/min with a column temperature of 60 °C. Mass spectrometry was performed in negative electrospray ionization mode with a nebulization gas pressure of 43.5 psi, dry gas flow rate of 12 L/min, a dry temperature of 250 °C, and a capillary voltage of 4000 V. Mass spectral data were collected over the range of 100–1500 m/z and auto calibrated using a 10 mM sodium formate solution at the end of data acquisition.

UHPLC–MS data processing took place using Bruker Metaboscape 5.0 (Bruker, Bellerica, MA, USA). Baseline noise intensity was set at 17,000 counts based on instrument response and interpretation of the baseline noise intensity throughout the chromatogram. Peaks were picked utilizing 10 scans forward and 5 scans recursively. Spectral library matching was performed with a m/z tolerance set at 5 ppm for narrow and 8 ppm for wide, mSigma of 10 for narrow and 30 for wide, and MS/MS score of 900 for narrow and 800 for wide. Analyte list m/z match tolerance was set at 2 ppm for narrow and 5 ppm for wide, RT match tolerance of 0.1 min for narrow and 0.8 min for wide, an mSigma of 10 for narrow and 20 for wide, and MS/MS score of 900 for narrow and 800 for wide. Data were normalized based to an umbelliferone internal standard. Confidence assignments for identified and unknown compounds, along with assignment of unknown nomenclatures, were performed in accordance with MSI recommendations [27]. Determination of isomeric compounds was performed using in-house libraries based on authentic standard measurements [43].

### 4.3. GC-Q-MS

Lyophilized border cell, secreted, root cap, elongation zone, and mature tissue samples were subjected to metabolite extraction using a biphasic solvent system [44]. Initially samples were combined with 500 µL of HPLC-grade chloroform, vortexed for 20 s, and sonicated for 20 s in a sonication bath. These samples were placed in a 50 °C oven for 1 h after which they were removed and combined with 500 µL of LC–MS-grade water. The samples were vortexed for 20 s and then placed in a 50 °C oven again for 1 h. Following the second 50 °C incubation, all samples were centrifuged at 4000× *g* for 1 h to separate the chloroform/water phases. 400 µL were removed from each phase to maintain consistency between samples. The upper water phase containing polar metabolites was transferred to a clean vial. The lower chloroform phase containing nonpolar metabolites was also transferred to a clean vial. Water and chloroform fractions were dried under a gentle stream of nitrogen gas. A solution containing internal standard was added to dried samples according to the polar or nonpolar samples as stated below.

Polar phase metabolites were resuspended using water containing 25 µg/mL ribitol, using 1 mL of water per 6 mg of starting dry tissue. These samples were again dried completely under nitrogen gas and then resuspended in 50 µL of fresh solution of pyridine containing 15 mg/mL methoxyamine-HCl. All samples were vortexed for 20 s and sonicated in a sonication bath for 10 s. Following sonication, all samples were transferred to a 50 °C oven for 1 h and then vortexed again. All samples were then combined with 50 µL of MSTFA + 1% TMCS, vortexed again for 20 s, and incubated at 50 °C for 1 h. Derivatized samples were removed from the oven, centrifuged briefly at 4000× *g* for 10 min, and transferred to an autosampler vial containing 150 µL vial insert.

Nonpolar phase metabolites were resuspended using chloroform containing 10 µg/mL docosanol, using 1 mL of chloroform per 6 mg of starting dry tissue. These samples were again dried down under nitrogen gas and then resuspended in 70 µL of pyridine by vortexing for 20 s. Resuspended samples were then combined with 30 µL of MSTFA + 1% TMCS, vortexed for 20 s, and then incubated at 50 °C for 1 h. Derivatized samples were centrifuged at 4000× *g* for 10 min and then transferred to an autosampler containing 150 µL vial insert.

Sample analyses were performed according to previously established protocol on an Agilent GC-MS (Agilent, Santa Clara, CA, USA) with a 6890N Network GC System and a 5973 Network Mass Selective Detector [44]. A 1 µL aliquot was injected using an Agilent ultra-inert wool 3390-2293 splitless liner (Agilent, Santa Clara, CA, USA) connected to an Agilent J&W GC Column (DB-5MS, 60 m length, 0.25 mm bore, 0.25 µm film) (Agilent, Santa Clara, CA, USA). Separations were achieved with a constant flow of 1.0 mL/min of helium gas and a temperature program of 80 °C for 2 min, then ramped at 5 °C/min to 315 °C and held at 315 °C for 12 min.

GC-MS peak picking, alignment, and quantification were done using AMDIS and MET-IDEA software. Data were normalized by dividing each peak area by the corresponding internal standard peak area, followed by dividing the total ion current intensity of each chromatogram. Metabolites were identified through accurate mass, and retention time matching with authentic compounds prepared and analyzed in an identical manner. Tentative identifications were performed by matching experimental mass data to those in plant metabolite databases and public literature. Confidence assignments for identified and unknown compounds, along with assignment of unknown nomenclatures, were performed in accordance with MSI recommendations [27].

### 4.4. Metabolite Atlas Development

A local in-house installation for the Arabidopsis Electronic Fluorescent Pictograph [24] was established and modified at the University of Missouri using publicly available source code http://www.bar.utoronto.ca/efp/development/ accessed on 28 August 2018. This source code was customized, developed further, and re-deployed to display metabolite data and imagery for *M. truncatula* roots using a similar heat map by tissue type organization. All Arabidopsis-related imagery was removed and replaced. This resulted in extensive modification of the webpage interface. Relative, normalized metabolite intensity data were uploaded into the locally hosted MySQL database along with control data values set to the baseline noise intensity. Metabolite relative abundance, for the purpose of this study, is defined by instrument response rather than a determined concentration. The *Medicago truncatula* Metabolite Atlas webpage was set up with search boxes for metabolite m/z ratio, International Chemistry Identifier (InChI) key, and metabolite name. As such, the background infrastructure was modified to search by m/z ratio, InChI Key, or metabolite name. It also incorporates autocomplete functionality using jQuery into the search boxes to show potential metabolites that match the search term being entered. The *Medicago truncatula* Metabolite Atlas is publicly available at http://artemis.cyverse.org/efp_medicago/cgi-bin/efpWeb.cgi.

## 5. Conclusions

A new Metabolite Atlas has been generated for the model legume *Medicago truncatula* that enables the visualization of the spatial distribution of metabolite relative abundances. This was achieved through successful spatial dissection of *Medicago truncatula* roots and analyses using UHPLC-QTOF-MS along with GC-Q-MS. Our data demonstrate that many metabolites exhibit differential accumulation and spatial localization when comparing across the mature tissue, the elongation zone, the root cap, border cells, and secreted fraction. While we have provided in depth analysis of a few specific metabolites, these are not intended to be a full representation of all metabolites that exhibit spatial localization within root regions. To better compile and visualize this data, we assembled the *Medicago truncatula* Metabolite Atlas as a public data repository. This metabolite atlas serves to show metabolite spatial localization in a heat map-based system using contextual imagery that is representative of the root fractions analyzed in our studies. Ultimately, this metabolite atlas eases the process of viewing metabolite spatial localization while providing a knowledge base for future analyses focused on the functional characteristics of spatially localized metabolites. As a public data repository, the *Medicago truncatula* Metabolite Atlas is envisioned as a dynamic database. However, continued development will depend on user support and future funding. We hope to include contributions to and utilization by other laboratories with the aim of creating synergistic connections and ultimately research collaborations.

## Data Availability

All data and the *Medicago truncatula* Metabolite Atlas are publicly available at http://artemis.cyverse.org/efp_medicago/cgi-bin/efpWeb.cgi.

## Supplementary Materials

The following are available online at https://www.mdpi.com/article/10.3390/metabo11040238/s1, Table S1. LC–MS root sectioning data. Table S2. GC-MS root sectioning data. Figure S1. Visualization of GC-MS metabolites in the *Medicago truncatula* Metabolite Atlas. Figure S2. Biosynthetic pathways of phenylalanine and tryptophan (KEGG). Figure S3. Biosynthetic pathway of 7,4′-dihydroxyflavone (DHF) (KEGG).
