# Supplementary material for: A Medicago truncatula Metabolite Atlas Enables the Visualization of Differential Accumulation of Metabolites in Root Tissues
Source: Metabolites. 2021 Apr 13;11(4):238. doi: 10.3390/metabo11040238 (PMC8068785; doi:10.3390/metabo11040238)
Supplement: Supplementary file 1 [file metabolites-11-00238-s001.zip › SupplementalFigS1-S3_Revised-10April2021.pdf]

Supplemental Figures S1-S3

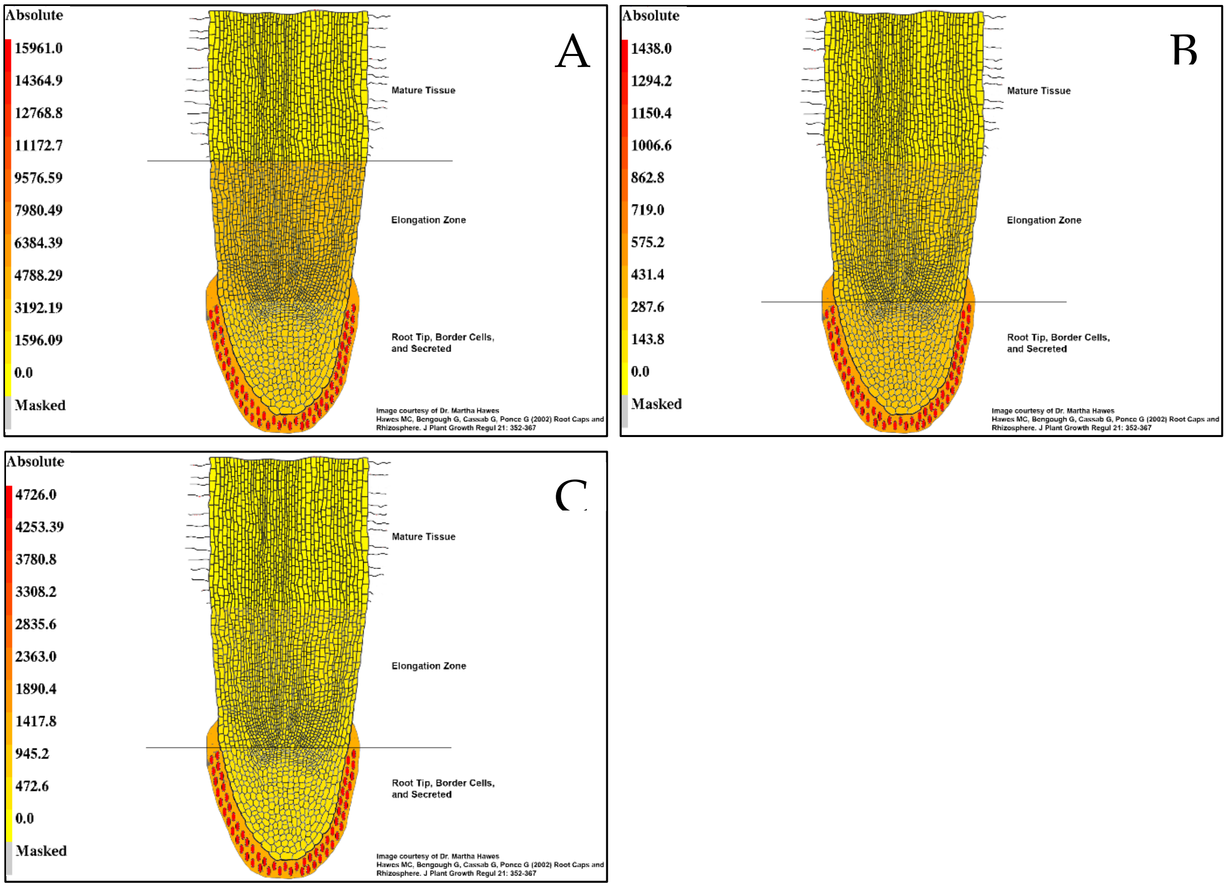

Figure S1. Visualization of GCMS Metabolites in the *Medicago truncatula* Metabolite Atlas. Regional comparison of (A) malic acid, (B) citric acid, and (C) succinic acid showed localization of metabolites in different tissues. The data indicate that some metabolites are localized in discrete root regions.

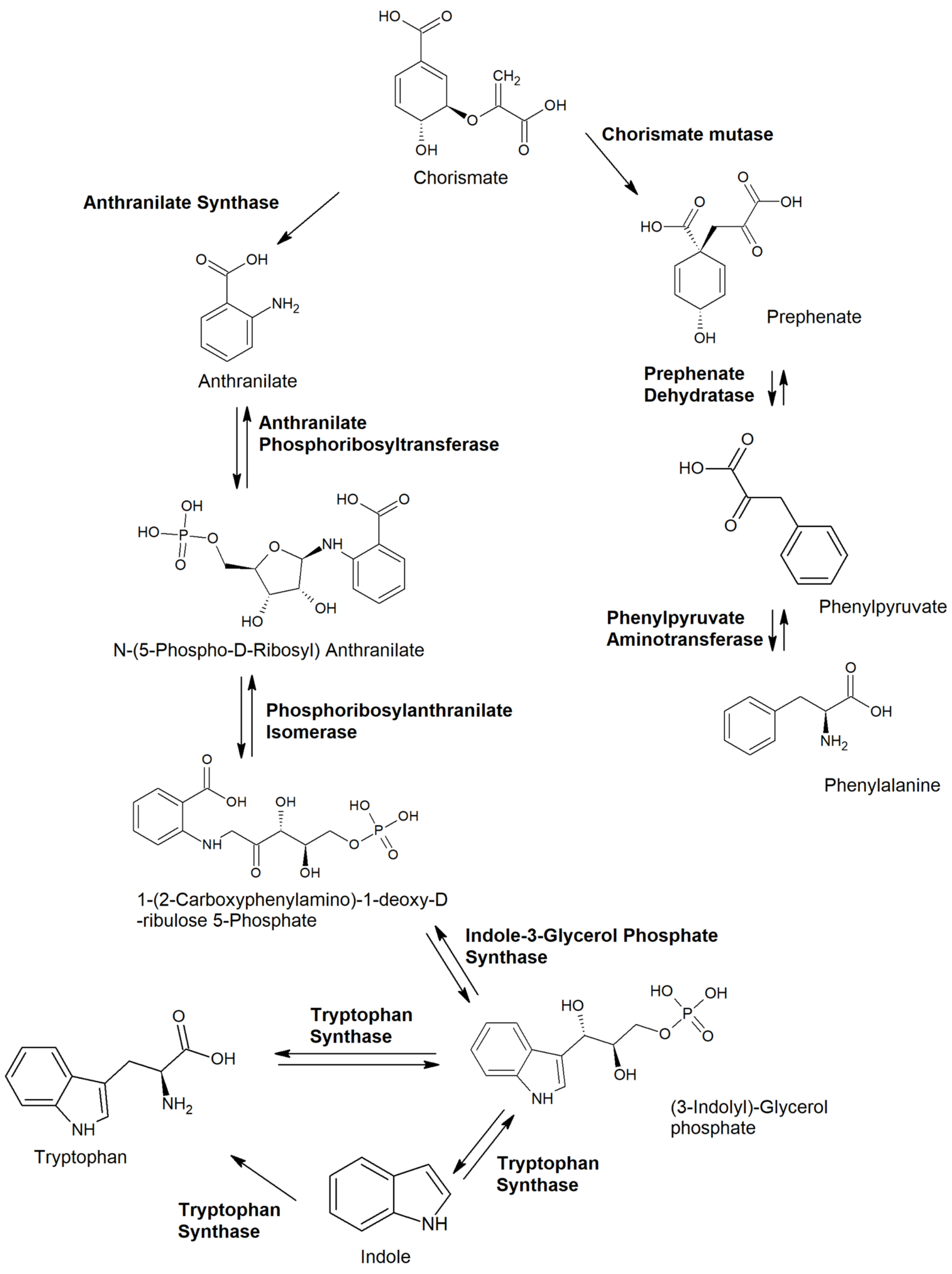

Figure S2. Biosynthetic pathways of phenylalanine and tryptophan (KEGG). Both phenylalanine and tryptophan showed a high degree of spatial localization in root fractions and play important roles in secondary metabolism and plant-microbe interactions. Biosynthesis of phenylalanine is in accordance with a recently discovered, primarily cytosolic pathway.

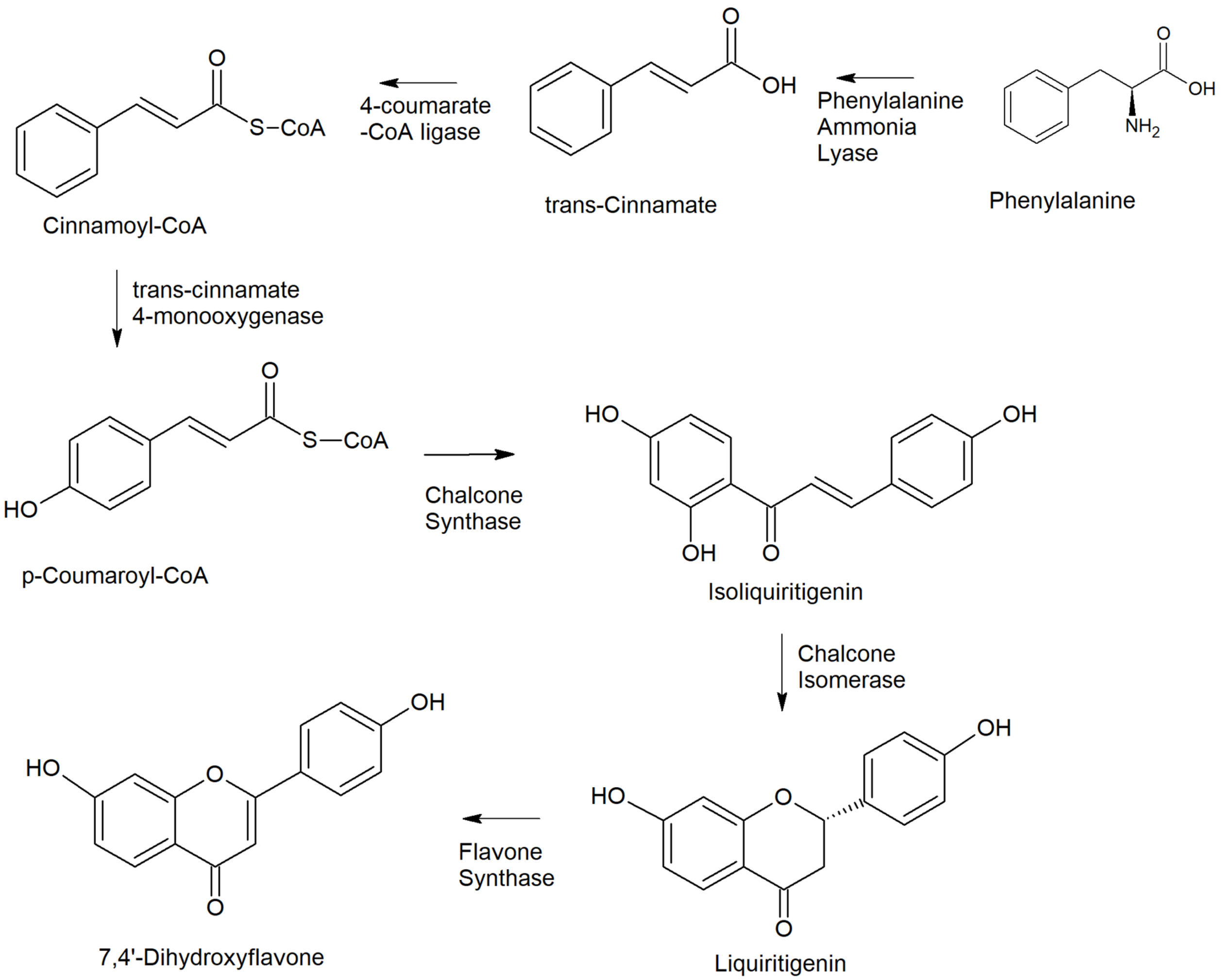

Figure S3. Biosynthetic pathway of 7,4′-dihydroxyflavone (DHF) (KEGG). DHF synthesis associated enzyme transcripts were reported to be upregulated in border cells and DHF showed a high degree of localization in border cells and the secreted fraction [4]. This compound is important in facilitating host-microbe interactions and plant defense.
